# Role of Matsuda Index in Identifying Patients at Risk for Cystic Fibrosis-Related Diabetes Development

**DOI:** 10.3390/children12111566

**Published:** 2025-11-18

**Authors:** Serpil Albayrak, Elif Arık, Özlem Keskin, Murat Karaoğlan, Mehmet Keskin, Gaye İnal, Mahmut Cesur, Ercan Küçükosmanoğlu, Ahmet Yıldırım

**Affiliations:** 1 Department of Pediatric Endocrinology, Gaziantep Cengiz Gökçek Maternity and Children’s Hospital, 27010 Gaziantep, Türkiye; 2 Department of Pediatric Allergy and Immunology, Faculty of Medicine, Gaziantep University, 27310 Gaziantep, Türkiye; drelif_tepe86@hotmail.com (E.A.); okeskin02@yahoo.com (Ö.K.); drgayeinal@hotmail.com (G.İ.); mahmutcesur@yahoo.com (M.C.); ercankosmanoglu@yahoo.com (E.K.); 3 Department of Pediatric Endocrinology, Faculty of Medicine, Gaziantep University, 27310 Gaziantep, Türkiye; muratkaraoglan@hotmail.com (M.K.); mkeskin@gantep.edu.tr (M.K.); ahmet161720@gmail.com (A.Y.)

**Keywords:** cystic fibrosis-related diabetes, diagnosis, HOMA-IR, Matsuda Index

## Abstract

**Highlights:**

**What are the main findings?**

- The Matsuda Index identified insulin resistance in 22% of pediatric cystic fibrosis patients, with significantly elevated postprandial insulin levels at 60 and 120 min.
- Pubertal children exhibited lower Matsuda Index scores, reflecting a decline in insulin sensitivity during puberty.

**What are the implications of the main findings?**

- Early detection of insulin resistance allows timely interventions (nutrition, physical activity, closer glucose monitoring) that may delay the onset of cystic fibrosis-related diabetes (CFRD).
- Puberty is a metabolically vulnerable period; integrating dynamic tests like OGTT with Matsuda Index into routine screening may improve risk stratification and outcomes.

**Abstract:**

**Background:** Cystic fibrosis-related diabetes (CFRD) is a frequent comorbidity in individuals with cystic fibrosis (CF). While insulin secretion defects are the primary mechanism in CFRD pathophysiology, insulin resistance may contribute as an additional risk factor. Early detection of insulin resistance may help identify patients at higher risk for earlier CFRD development. **Objective:** The aim of this study was to evaluate the ability of the Matsuda Index to identify insulin resistance in pediatric CF patients and to compare it with HOMA-IR as complementary indicators of glucose metabolism. **Methods:** In this cross-sectional study, fifty children with CF aged 6–16 years were included. The study involved measuring anthropometric data, fasting insulin, fasting glucose levels, glycated hemoglobin (HbA1c), and C-peptide. An assessment of glucose and insulin levels was performed on the patients through an oral glucose tolerance test (OGTT) at 0, 60, and 120 min. The Matsuda Index was computed, wherein a threshold of ≤4.5 signifies the presence of insulin resistance. Statistical analyses were conducted to compare insulin resistance and sensitivity across groups, using *t*-tests, correlation, and ANOVA. **Results**: Among the 50 observed patients, the average Matsuda index score was 17.08 with a standard deviation of 11.16. Eleven individuals (22%) exhibited insulin resistance with a Matsuda Index ≤ 4.5. These patients showed significantly higher insulin levels at 60 and 120 min during the OGTT, with statistically significant *p*-values of 0.008 and 0.002, respectively. **Conclusions:** The Matsuda Index may serve as a useful adjunctive tool to help identify insulin resistance in pediatric CF patients, particularly during puberty. Early detection of insulin resistance through the Matsuda Index may facilitate risk stratification and enable timely interventions that could potentially delay the onset or progression of CFRD. However, it should be noted that the ≤4.5 cut-off value was derived from adult studies, and its validity in pediatric CF populations has not been established, which represents a limitation of our finding.

## 1. Introduction

Cystic fibrosis (CF) is a complex genetic disorder that poses life-threatening risks to multiple organs, including the lungs, pancreas, liver, and intestines. Cystic fibrosis-related diabetes (CFRD) is a significant comorbidity often found in patients with cystic fibrosis [1]. While insulin secretion defects are the primary mechanism in CFRD pathophysiology, insulin resistance may act as an additional risk factor that accelerates CFRD development. The identification of concurrent insulin resistance may provide important clinical information for risk stratification [2,3].

Insulin resistance in cystic fibrosis patients serves as an important contributing factor to CFRD development, in addition to insulin secretion defects. Direct effects of CFTR protein deficiency result in impaired glucose-induced insulin secretion and pancreatic β-cell dysfunction [4]. Furthermore, pro-inflammatory factors such as IL-6 secreted from pancreatic ductal cells in CFTR-deficient states negatively affect islet insulin secretion through paracrine mechanisms [5]. Glucose Transporter (GLUT)-4 translocation is impaired in CF patients, leading to reduced peripheral glucose uptake [6]. The chronic systemic inflammatory process, particularly elevated TNF-α levels, contributes to the development of insulin resistance by decreasing insulin sensitivity [6]. This multifactorial process becomes more pronounced during puberty due to the effects of growth hormone and sex steroids [7,8].

Chronic inflammation often intensifies insulin resistance, while pancreatic islet cell damage impairs insulin secretion, together characterizing CFRD. The presence of inflammation and the frequent administration of corticosteroids in CF patients play a significant role in the development of insulin resistance. Glucose metabolism is further complicated by malnutrition and metabolic disturbances [2]. Although CFRD is uncommon and silent in childhood, its prevalence increases with age, affecting 21% of children aged 10 years and younger, 19% of those aged 10–17 years, and 30% to 50% of adult cystic fibrosis patients [3,4].

Common approaches, such as the oral glucose tolerance test (OGTT), are still considered the gold standard for diabetes detection [5]. Nevertheless, the intricate nature of glucose metabolism in CFRD warrants a more nuanced approach to assessing metabolic control. As a result, there has been an increasing interest in indices, such as the Homeostatic Model Assessment of Insulin Resistance (HOMA-IR) and the Matsuda Index. These indices have the potential to identify metabolic disturbances in CF patients at an earlier stage [6,7]. The entire insulin response during the OGTT, the Matsuda Index becomes a valuable tool for identifying even subtle changes in insulin sensitivity, making it especially useful in diseases like CFRD where both insulin secretion and resistance are affected [8]. Nevertheless, in pediatric CF, the Matsuda Index should be regarded as a potential predictor rather than a validated risk factor since pediatric-specific validation studies are still lacking.

The Matsuda Index uses OGTT data, including fasting and postprandial glucose and insulin levels, to assess insulin sensitivity in children. Early detection of insulin resistance in children is vital for the prevention of future metabolic disorders [9,10]. A lower index score is linked to a greater risk of diabetes progression in relatives with autoantibodies [10]. In children, the Matsuda Index correlates more closely with insulin sensitivity, making it potentially superior for pediatric assessments. Compared to HOMA-IR, the Matsuda Index offers a more reliable assessment of metabolic health, allowing for timely interventions targeted at pediatric patients [8,11].

Extensive studies have confirmed the validity of this threshold, showing its potential as a screening tool for CFRD patients. Moreover, the Matsuda Index has demonstrated a robust association with insulin levels throughout various stages of the OGTT, offering a more precise depiction of insulin sensitivity across diverse temporal intervals [12]. Early detection of insulin resistance or impaired insulin secretion, even before hyperglycemia, permits timely interventions, such as lifestyle modifications or pharmacological treatments, that can effectively delay or prevent the onset of CFRD [13].

The objective of this study was to evaluate whether the Matsuda Index can accurately identify insulin resistance among pediatric cystic fibrosis patients and to compare its performance with HOMA-IR, hypothesizing that the Matsuda Index would better capture early metabolic alterations predisposing to CFRD.

## 2. Materials and Methods

### 2.1. Study Design

A cross-sectional observational study was conducted to investigate the glucose metabolism of CF patients and identify early indicators of CFRD. Children with CF, who were at least 6 years old, were selected for the study. As part of their routine clinical follow-up, they underwent an OGTT.

A total of 62 eligible patients were screened, and 50 met the inclusion criteria after excluding those with known CFRD. A total of 50 pediatric CF patients were consecutively included during their routine follow-up visits to minimize potential selection bias. The patients were recruited from a specialized tertiary care center on managing cystic fibrosis. To participate in the study, individuals were required to meet the following criteria: a confirmed diagnosis of cystic fibrosis, an age of at least six years, and prior completion of an oral glucose tolerance test. Patients diagnosed with any other type of diabetes, including type 1 or type 2 diabetes, were excluded from the study. Patients who were prescribed medications that have a notable effect on glucose metabolism, such as insulin or corticosteroids, within the previous 12 months were excluded. No healthy or obese control group was included, as the study focused on within-CF metabolic variability.

The study was conducted in accordance with the Declaration of Helsinki and received institutional review board approval (Approval number: 2023/439, date: 12 April 2023). All participants’ parents or legal guardians provided written informed consent, and children over the age of 12 also gave their assent. The study maintained patient anonymity and confidentiality by implementing measures such as limiting data collection and allowing access only to the study investigators.

### 2.2. Data Collection and Measurements

For every patient, precise anthropometric measurements, including weight, height, and body mass index (BMI), were recorded. National reference growth charts were utilized to calculate standard deviation scores (SDS) for weight-for-age and height-for-age. We also collected fasting blood samples to measure glucose, insulin, C-peptide, and glycated hemoglobin (HbA1c). Vitamin D levels were measured during routine winter and spring follow-up visits; seasonal variation might have influenced the observed mean levels.

An OGTT was conducted for each patient following an overnight fasting. During the test, patients consumed 1.75 g/kg of glucose (with a maximum limit of 75 g). Glucose and insulin levels were measured at 0, 60, and 120 min after glucose ingestion. In contrast with the standard Matsuda Index formula, which also requires a 30 min sample, our calculation relied only on 0, 60, and 120 min values due to retrospective data availability. This modification may slightly underestimate early insulin responses and, consequently, overestimate overall insulin sensitivity compared with the standard five-point OGTT. Similar modified sampling methods have been applied in pediatric metabolic studies [7]. This methodological limitation should be considered when interpreting our results.

A diagnosis of CFRD is confirmed when fasting blood glucose levels are equal to or greater than 126 mg/dL (7 mmol/L) or 2 h postprandial blood glucose levels are equal to or greater than 200 mg/dL (11.1 mmol/L), persist for at least 48 h, and have 2 or more elevated blood glucose values [14]. CFTR genotype information was retrieved from medical records when available; however, detailed mutation-specific analyses were not performed due to incomplete data.

The Matsuda Index is a widely utilized tool for assessing insulin sensitivity, relying on glucose and insulin measurements acquired during an OGTT. It includes both fasting and post-meal glucose and insulin levels, measured at intervals of 30, 60, 90, and 120 min after eating. The formula for the Matsuda Index is presented below:

$$\mathrm{Matsuda}\;\mathrm{Index}=\frac{10,000}{\sqrt{\left(\mathrm{FPG}\times \mathrm{FPI}\right)\times \left(\mathrm{Mean}\;\mathrm{Glucose}\times \mathrm{Mean}\;\mathrm{Insulin}\right)}}$$

Matsuda Index (calculated using the WEB calculator).

The Matsuda Index was calculated using an online calculator.

Matsuda Index Threshold Selection: While various threshold values for the Matsuda Index have been reported in the literature, we selected a threshold of ≤4.5 based on studies demonstrating its utility in identifying insulin resistance across different populations [11]. This threshold, however, was adopted from adult cohorts and has not been specifically validated in pediatric CF populations, underscoring the need for age- and disease-specific cut-off studies [12].

### 2.3. Statistical Analysis

By employing the Tanner criteria for pubertal staging assessment, patients were classified into prepubertal and pubertal groups. Potential confounding factors such as acute infections, recent hospitalizations, or malnutrition that could interfere with glucose metabolism were excluded, and patients were stratified by pubertal stage and BMI SDS to minimize bias. This stratification was performed to explore the potential influence of pubertal changes on glucose metabolism. Additionally, the cohort was divided into two groups based on their Matsuda Index values (≤4.5 and >4.5) to examine disparities in insulin sensitivity and resistance.

Continuous variables were summarized as median (IQR) or mean ± SD as appropriate. Categorical variables were presented as n (%). Group comparisons were performed using the Mann–Whitney U or independent t-tests for continuous variables, and Fisher’s exact test for categorical variables. Differences across pubertal status were analyzed similarly. We evaluated the effect of using a modified (0–60–120 min) Matsuda Index versus the standard (0–30–60–90–120 min) formula by performing a paired *t*-test and quantifying agreement with the κ statistic. A sensitivity analysis using the 25th percentile (10.97) as a pediatric-specific cut-off was performed. Statistical significance was set at *p* < 0.05. All analyses were performed using IBM SPSS Statistics v26 (IBM Corp., Armonk, NY, USA).

## 3. Results

A total of 50 pediatric patients diagnosed with CF were selected for this study, with 23 females (46%) and 27 males (54%) forming the cohort. The cohort was evenly distributed by sex, enabling meaningful sex-based comparisons. Patients were aged 6–16 years, with a mean age 11.24 ± 2.64 years. The average disease duration for all participants was 11.24 ± 2.67 years. Therefore, the sample was divided into two groups: prepubertal (48%, n = 24) and pubertal (52%, n = 26). None of the participants met the diagnostic criteria for CFRD during the study period.

The HbA1c level exhibited variation, with an average of 5.39 ± 0.47%. Variations in insulin production capacity were observed among patients, with an average C-peptide level of 1.38 ± 1.12 ng/mL.

Glucose values were measured during the OGTT. The average fasting glucose level was determined to be 77.46 ± 12.93 mg/dL. The average glucose level at 60 min was 132.54 ± 56.49 mg/dL, while at 120 min, it was 110.42 ± 64.68 mg/dL. Similarly, insulin levels were a mean fasting insulin level of 4.47 ± 3.14 µU/mL, a mean insulin level at 60 min of 25.13 ± 19.77 µU/mL, and a mean insulin level at 120 min of 22.05 ± 24.66 µU/mL. Table 1 displays the descriptive statistics for the laboratory and clinical parameters of participants.

Based on anthropometric measurements, patients had a mean weight SDS (standard deviation score) of −1.30 ± 1.74, indicating that they were underweight for their age and sex. Patients’ actual weight varied between 18.4 kg and 62.0 kg, with a mean of 32.97 ± 9.78 kg. Based on the height SDS of −0.72 ± 1.59, it can be inferred that patients’ heights fell slightly below the expected range.

### Matsuda Index Groups

Among the 50 patients, 11 individuals (22%) showed insulin resistance, as shown by a Matsuda Index below 4.5. The remaining 39 patients (78%) demonstrated better insulin sensitivity with a Matsuda Index greater than 4.5. We conducted a comparative analysis of these groups to evaluate glucose and insulin levels at various time points during the OGTT (0, 60, and 120 min). An average Matsuda Index of 17.08 ± 11.16 was observed in the entire group. There was no notable variation in C-peptide levels (*p* = 0.485) between the groups.

**Figure 1:**
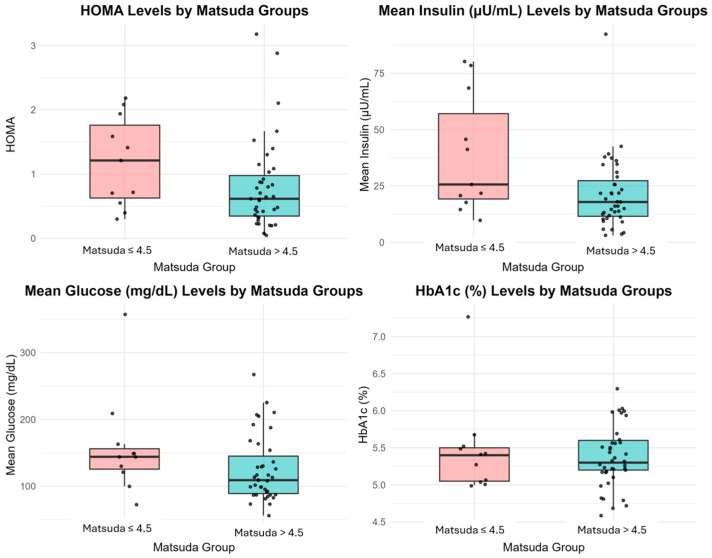
Comparative box plots of HOMA-IR, Insulin, Glucose, and HbA1c levels between Matsuda ≤ 4.5 and >4.5 groups. Each box represents the interquartile range (IQR) with the horizontal line indicating the median value; whiskers extend to 1.5 × IQR, and individual points represent outliers.

In patients with a Matsuda Index ≤ 4.5, the insulin levels at 60 and 120 min during OGTT were significantly higher than those with values > 4.5 (*p* = 0.008 and *p* = 0.002, respectively). In particular, the insulin level at 60 min was recorded as 38.60 ± 26.25 µU/mL in the group with insulin resistance, in contrast to 21.34 ± 15.96 µU/mL in the group without resistance. The insulin levels in the insulin-resistant group remained elevated at 120 min, averaging 30.99 ± 38.22 µU/mL, in contrast to the non-resistant group’s average of 19.53 ± 19.24 µU/mL. Median (IQR) HOMA-IR values were 1.67 (1.21–1.94) in the insulin-resistant group and 0.48 (0.40–1.21) in the insulin-sensitive group (*p* = 0.136), showing a non-significant trend toward higher insulin resistance (Figure 1).

Although there were notable variations in insulin levels, there was no statistically significant disparity observed in fasting glucose levels between the Matsuda Index groups (*p* = 0.450). However, patients with a Matsuda Index ≤ 4.5 exhibited increased glucose levels at 60 and 120 min compared to individuals with higher Matsuda values. The differences observed did not achieve statistical significance, with *p*-values of 0.091 and 0.623 for each, respectively. At 60 min, the insulin-resistant group showed a mean glucose level of 158 ± 74.63 mg/dL, whereas the non-resistant group had 125 ± 49.08 mg/dL. Correspondingly, the mean glucose level in the insulin-resistant group was 119 ± 102.26 mg/dL at the 120 min mark, while the non-resistant group showed a level of 108 ± 51.15 mg/dL. Detailed results of the Matsuda Index groups are presented in Table 2.

When analyzed by pubertal status, the mean Matsuda Index was significantly lower in pubertal participants (15.86) compared to prepubertal children (10.44, *p* < 0.048). The proportion of patients with Matsuda ≤ 4.5 was higher in the pubertal group (30.8% vs. 12.5%, *p* = 0.045). These findings are summarized in Table 3.

In the bias analysis comparing the three-point and standard five-point Matsuda Index, the mean difference was 0.83 ± 0.86 (*p* < 0.001), with only 2% reclassification and a good agreement (κ = 0.79). Using the 25th percentile value (10.97) as an alternative pediatric-specific cut-off resulted in 22% reclassification and poor agreement (κ = 0.21), indicating that the adult-derived ≤4.5 threshold may underestimate insulin resistance prevalence in this cohort (Table 4).

## 4. Discussion

In this study, we aimed to evaluate the role of the Matsuda Index in identifying CF patients at risk for CFRD development. The Matsuda Index, originally developed for adults, has been widely used to evaluate insulin sensitivity. In our cohort, approximately 22% of patients were classified as insulin-resistant according to the ≤4.5 threshold. Patients with a Matsuda Index ≤ 4.5 exhibited higher glucose levels and lower insulin sensitivity. Pubertal children demonstrated lower Matsuda Index values than prepubertal participants. This difference may be explained by physiological insulin resistance that occurs during puberty. The absence of the 30 min OGTT point may have slightly underestimated early insulin responses and, consequently, overestimated the overall insulin sensitivity. CFRD is a frequent condition that occurs alongside CF, and its occurrence becomes more common as individuals with CF get older [13]. The insulin resistance observed in CF patients results from a complex and multifactorial process. In our study, patients with insulin resistance demonstrated elevated insulin levels characterized by compensatory hyperinsulinemia. This indicates that the pancreas produces higher amounts of insulin in response to increasing glucose levels [15]. However, the long-term sustainability of this compensatory mechanism remains uncertain, creating a potential risk for CFRD development as pancreatic β-cell function gradually deteriorates [16,17]. This relationship may partly explain why the early detection of insulin resistance is essential for timely intervention in CF. The early identification of insulin resistance in CF patients through the Matsuda Index enables several targeted interventions that may delay or prevent CFRD onset. Nutritional optimization allows for specialized nutritional counseling focusing on carbohydrate management and maintaining optimal body weight, which is crucial in CF patients who are often underweight [18]. Regular physical activity has been shown to improve insulin sensitivity in various populations, including CF patients, enabling the implementation of structured exercise programs tailored to the patient’s pulmonary function [19].

A key finding was the lower Matsuda Index in pubertal versus prepubertal participants, consistent with previous reports showing physiologic insulin resistance during puberty. These findings align with the documented physiological changes that occur during puberty, wherein elevated levels of growth hormone, insulin-like growth factor-1 (IGF-1), and sex steroids contribute to a temporary state of insulin resistance [16,18]. This underscores the significance of conducting thorough metabolic monitoring of CF patients during puberty, as they may be at a heightened risk for early CFRD development [20]. These observations collectively emphasize the need for age-specific screening strategies and close metabolic follow-up during puberty. These findings highlight puberty as a critical window for metabolic monitoring in CF. Another limitation is the absence of diagnostic performance analyses (e.g., ROC curves) comparing the Matsuda Index with other screening tools, such as HOMA-IR, which would have strengthened the evidence of its clinical utility.

Furthermore, the increased insulin resistance during puberty emphasizes the necessity for age-tailored screening protocols in individuals with CF. According to the current guidelines, it is recommended to screen for CFRD at the age of 10 [1,21]. Our research suggests that screening during puberty, when metabolic vulnerability is heightened, should be intensified. Routine screening should include dynamic measures, such as the OGTT and Matsuda Index, as fasting measures like HOMA-IR and HbA1c may not be sufficiently sensitive to detect early metabolic disturbances. The utilization of postprandial evaluations, specifically the Matsuda Index, might prove to be more effective in pinpointing initial impairments in glucose metabolism within this specific population. Taken together, these glucose and insulin dynamics support the use of the Matsuda Index as a sensitive marker of early insulin resistance in CF. The clinical relevance of this finding lies in its support for integrating dynamic tests like the OGTT, along with indices like the Matsuda Index, to improve the accuracy of CFRD screening and diagnosis [6,22].

Although there were no significant differences in fasting glucose levels between the groups, patients with a Matsuda Index of 4.5 or lower showed higher glucose levels 60 and 120 min after consuming glucose. The importance of monitoring glucose levels throughout the OGTT, rather than just fasting glucose levels, is underscored by this finding, as it helps identify early disturbances in glucose metabolism [20]. What was even more noticeable were the stark disparities in insulin levels observed between the two groups. Insulin levels at 60 and 120 min exhibited a notable increase in the insulin-resistant group. Nonetheless, this compensatory hyperinsulinemia may impose further stress on pancreatic β-cells, resulting in β-cell depletion and subsequent insulin insufficiency, characteristic of CFRD [23]. Elevated insulin levels in the insulin-resistant group suggest β-cell dysfunction [24]. HbA1c levels were similar in insulin-resistant and non-insulin-resistant groups, despite variations in insulin and glucose dynamics during the OGTT. Although additional biochemical parameters such as calcium, phosphorus, ALP, PTH, vitamin D, magnesium, and TSH were measured, they were collected as part of routine CF follow-up and were not directly related to the study hypothesis; therefore, they were not included in comparative analyses. To maintain focus, these data were not emphasized in the main analysis and may be considered supplementary information if needed. In CF patients, particularly those with fluctuating glucose levels or temporary hyperglycemia, HbA1c may not possess sufficient sensitivity to identify early glucose metabolism disturbances. This finding supports that HbA1c has limited effectiveness in detecting early CFRD [25,26]. It emphasizes the necessity for more dynamic assessments like OGTT and indices, such as the Matsuda Index, to identify early metabolic abnormalities [27]. Collectively, these findings reinforce the role of dynamic tests in detecting subclinical metabolic changes. There were no significant differences in C-peptide levels between the groups. This observation suggests that despite the insulin resistance in certain patients, the capacity to produce insulin remains relatively unaffected in both groups.

The findings of this research carry crucial clinical implications for the management of glucose metabolism in pediatric CF patients. The Matsuda Index correlates with insulin sensitivity and may be useful for detecting insulin resistance in CF patients [12]. Early identification of high-risk patients allows interventions to delay diabetes onset [2].

Puberty’s significant contribution to increased metabolic risk and insulin resistance is evident in our research and that of Kurtoglu et al. [28]. In contrast with the cited study, the Matsuda Index offers superior utility in the diagnosis of insulin resistance among cystic fibrosis patients. A total of 22% of our cohort showed significant insulin resistance (Matsuda ≤ 4.5), especially during puberty.

Lastly, the study emphasizes the significance of longitudinally tracking glucose and insulin dynamics in patients diagnosed with cystic fibrosis, specifically those with borderline Matsuda Index values. Routine monitoring enables the detection of the shift from insulin resistance to CFRD, facilitating prompt interventions that can enhance long-term outcomes. Nonetheless, our study was based on a relatively small, single-center cohort of 50 children, with only 11 identified as insulin resistant, which limits the generalizability of the findings.

While the Matsuda Index shows promise as a screening tool, certain methodological aspects warrant caution. Considering the overall findings, certain methodological and population-related limitations must also be recognized. The cross-sectional design precludes establishing causality between insulin resistance, as measured by the Matsuda Index, and the eventual development of CFRD. Longitudinal studies are required to determine whether patients with early insulin resistance progress more rapidly to CFRD. The threshold value of ≤4.5 for the Matsuda Index was derived from studies conducted primarily in adult populations, and validation in pediatric CF cohorts is lacking. Therefore, the observed 22% prevalence of insulin resistance may represent an underestimation of the true rate in children. Future research should establish pediatric- and disease-specific cutoff values. HbA1c was measured at a single time point; longitudinal averages were unavailable, representing a limitation. Our relatively small, single-center cohort (n = 50, with only 11 cases of insulin resistance) limits the generalizability of the findings, underscoring the need for larger multicenter studies. We did not perform formal diagnostic accuracy analyses (e.g., ROC curves) comparing Matsuda with other indices, and potential confounders such as BMI SDS, nutritional status, inflammation, and pubertal stage were not adjusted beyond simple stratification. Because confirmatory validation tests such as euglycemic–hyperinsulinemic clamps or ROC curve analyses were not performed, our conclusions should be interpreted as exploratory rather than confirmatory. The absence of a healthy control or exogenous obesity group limits the external validity of our results and precludes direct comparison of Matsuda Index thresholds with those established in non-CF or obese pediatric populations. Finally, while our study demonstrates the presence of insulin resistance, it should be interpreted as a contributory rather than primary factor in CFRD pathophysiology, and whether early detection translates into improved long-term outcomes remains to be determined through prospective intervention studies.

## 5. Conclusions

This study suggests that the Matsuda Index may be a valuable adjunct for identifying insulin in pediatric CF patients, with 22% of our cohort showing significant insulin resistance (Matsuda Index ≤ 4.5). While insulin secretion defects remain the primary mechanism in CFRD pathophysiology, the identification of concurrent insulin resistance may provide important clinical information for risk stratification. The Matsuda Index emerges as a useful screening tool that when combined with standard OGTT, can help identify CF patients who may be at higher risk for earlier CFRD development, particularly during the metabolically vulnerable period of puberty. Early identification of insulin resistance enables the implementation of targeted interventions that may potentially delay the onset or slow the progression of CFRD. Prospective longitudinal studies are needed to validate the prognostic value of the Matsuda Index in predicting CFRD development and to establish optimal threshold values specifically for pediatric CF populations. Additionally, intervention studies are required to determine whether early treatment of insulin resistance in CF patients improves long-term metabolic outcomes and delays CFRD onset.

## Figures and Tables

**Table 1:** Descriptive Statistics for Demographic, Anthropometric, and Laboratory Parameters.

Characteristic	Value (n = 50)	IQR
**Demographic Parameters**		
**Sex**		
*Female*	23 (46%)	
*Male*	27 (54%)	
**Pubertal Status**		
*Prepubertal*	24 (48%)	
*Pubertal*	26 (52%)	
**Anthropometric Measures**		
*Weight SDS*	−1.13 (−2.52–0.01)	2.53
*Height SDS*	−1.27 (−2.28–−0.15)	2.13
**Laboratory Measurements**		
**HbA1c (%)**	5.39 ± 0.47	
**C-peptide (ng/mL)**	1.04 (0.78–1.66)	0.88
**Glucose (mg/dL)**		
*Fasting*	76.50 (69.00–84.00)	15.00
*60 min*	117.00 (92.00–154.00)	62.00
*120 min*	95.00 (87.00–114.00)	27.00
**Insulin (µU/mL)**		
*Fasting*	3.85 (2.15–6.14)	3.99
*60 min*	20.02 (12.54–34.48)	21.94
*120 min*	13.31 (5.99–22.37)	16.38
**HOMA-IR**	0.68 (0.40–1.21)	0.81
**Matsuda Index Score**	11.29 (6.13–18.00)	11.87
**DEXA (T-score)**	−0.90 (−2.20–0.00)	2.20
**Calcium (mg/dL)**	9.77 ± 0.48	
**Phosphorus (mg/dL)**	4.40 ± 0.49	
**ALP (U/L)**	225.00 (199.00–300.00)	101.00
**PTH (pg/mL)**	49.30 (32.70–64.70)	32.00
**Vitamin D (ng/mL)**	20.35 (12.26–24.18)	11.92
**Magnesium (mg/dL)**	1.99 ± 0.14	
**TSH (µU/mL)**	2.32 (1.73–2.95)	1.23

Continuous variables were denoted in the Mean ± SD (Standard Deviation) value format. Categorical variables were expressed as n (%). SDS: Standard Deviation Score, DEXA: Dual-energy X-ray absorptiometry, ALP: Alkaline Phosphatase, PTH: Parathyroid Hormone, TSH: Thyroid-stimulating Hormone, T4: Thyroxine Test.

**Table 2:** Comparative Analysis of Insulin and Glucose Levels by the Matsuda Index Group.

	Matsuda Index Group		*p*
	≤4.5 (n = 11)	>4.5 (n = 39)	
**Glucose (mg/dL)**			
*Fasting*	76 (70–87)	77 (68–84)	0.450
*60 min*	144 (121–163)	09 (87–154)	0.091
*120 min*	94 (87–144)	95 (87–114)	0.632
**Insulin (µU/mL)**			
*Fasting*	3.85 (2.15–6.14)	3.99 (2.15–6.14)	0.112
*60 min*	38.6 (26.3–74.6)	21.3 (16.0–49.1)	0.008 *
*120 min*	31.0 (19.5–38.2)	19.5 (19.2–19.2)	0.002 *
**C-peptide**	1.60 (1.28–1.66)	1.33 (1.08–1.08)	0.485
**HbA1c**	5.47 (0.65)	5.37 (0.42)	0.516
**HOMA-IR**	1.67 (0.48–1.21)	0.48 (0.40–1.21)	0.136

Continuous variables were denoted in the Mean ± SD (Standard Deviation) value format. Statistical significance was indicated by the * sign for the *p*-values < 0.05.

**Table 3:** Comparison of Insulin Sensitivity and Glycemic Parameters Between Prepubertal and Pubertal Cystic Fibrosis Patients.

Variable	Prepubertal (n = 24)	Pubertal (n = 26)	*p*-Value
**Matsuda Index**	15.86 (8.26–21.47)	10.44 (4.22–12.76)	0.048 *
**Glucose 60 min (mg/dL)**	111 (91–156)	153 (99–154)	0.092
**Glucose 120 min (mg/dL)**	95 (89–104)	93 (86–116)	0.114
**Insulin 60 min (µU/mL)**	13.69 (9.58–24.52)	21.87 (16.09–37.40)	0.043 *
**Insulin 120 min (µU/mL)**	11.36 (4.83–14.18)	19.36 (9.06–29.21)	0.066
**C-peptide (ng/mL)**	0.98 (0.63–1.66)	1.09 (0.84–1.68)	0.218
**HOMA-IR**	0.46 (0.33–0.99)	0.84 (0.55–1.40)	0.071
**Matsuda ≤ 4.5 (%)**	12.50%	30.80%	0.045 *

Continuous variables were denoted in the Mean ± SD (Standard Deviation) value format. Statistical significance was indicated by the * sign for the *p*-values < 0.05.

**Table 4:** Statistical Agreement of Adult-Derived and Pediatric-Specific Matsuda Index Thresholds.

Analysis	Median Difference	*p*-Value	Reclassification Rate	κ
**3-point vs. 5-point Matsuda**	0.79	<0.001	2%	0.79
**25th percentile (10.97) vs. ≤4.5 cut-off**	—	—	22%	0.21

## Data Availability

The data supporting the findings are available from the corresponding author upon reasonable request. Data sharing is restricted by ethical approval conditions and institutional policies to protect participant confidentiality.

## Abbreviations

The following abbreviations are used in this manuscript:

CF	Cystic Fibrosis
CFRD	Cystic Fibrosis-Related Diabetes
CFTR	Cystic Fibrosis Transmembrane Conductance Regulator
OGTT	Oral Glucose Tolerance Test
HbA1c	Glycated Hemoglobin
SDS	Standard Deviation Score
BMI	Body Mass Index
HOMA-IR	Homeostatic Model Assessment of Insulin Resistance
DEXA	Dual-Energy X-ray Absorptiometry
ALP	Alkaline Phosphatase
PTH	Parathyroid Hormone
TSH	Thyroid-Stimulating Hormone
ROC	Receiver Operating Characteristic
